# Ethyl 2-{(2*Z*)-2-[(1-naphthyl­sulfon­yl)imino]-2,3-dihydro-1,3-thia­zol-4-yl}acetate monohydrate

**DOI:** 10.1107/S1600536810039279

**Published:** 2010-10-09

**Authors:** Gabriel Navarrete-Vázquez, Guadalupe Morales-Vilchis, Samuel Estrada-Soto, Verónica Rodríguez-López, Hugo Tlahuext

**Affiliations:** a Facultad de Farmacia, Universidad Autónoma del Estado de Morelos, Av. Universidad 1001 Col Chamilpa CP 62209, Cuernavaca Mor., México; b Centro de Investigaciones Químicas, Universidad Autónoma del Estado de Morelos. Av. Universidad 1001 Col., Chamilpa, CP 62209, Cuernavaca Mor., México

## Abstract

The title compound, C_17_H_16_N_2_O_4_S_2_·H_2_O, is of inter­est with respect to its anti­diabetic and anti-obesity activity. In the crystal, the packing is stabilized by three cooperative inter­actions: offset π–π inter­actions [centroid–centroid distance = 3.604 (2) Å], as well as C—H⋯O and O—H⋯O hydrogen bonds. N—H⋯O inter­actions also occur.

## Related literature

For similar structures and their anti­diabetic activity, see: Navarrete-Vázquez *et al.* (2008 ▶); Alberts *et al.* (2002 ▶); Barf *et al.* (2002 ▶); Fotsch & Wang (2008 ▶); Saiah (2008 ▶); Vicker *et al.* (2007 ▶). For hydrogen bonds, see: Adams *et al.* (1996 ▶); Desiraju & Steiner (1999 ▶); Hanton *et al.* (1992 ▶).
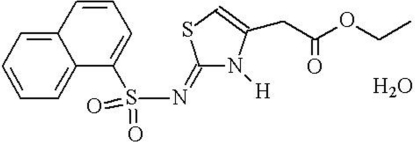


## Experimental

### 

#### Crystal data

C_17_H_16_N_2_O_4_S_2_·H_2_O
*M* *_r_* = 394.45
Orthorhombic, 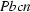

*a* = 29.582 (6) Å
*b* = 7.9657 (17) Å
*c* = 15.676 (3) Å
*V* = 3694.0 (14) Å^3^
*Z* = 8
Mo *K*α radiation
μ = 0.32 mm^−1^
*T* = 273 K
0.29 × 0.21 × 0.17 mm

#### Data collection

Bruker SMART APEX CCD area detector diffractometer
Absorption correction: multi-scan (*SADABS*; Sheldrick, 1996 ▶) *T*_min_ = 0.913, *T*_max_ = 0.948
33131 measured reflections
3255 independent reflections
2488 reflections with *I* > 2σ(*I*)
*R*_int_ = 0.056

#### Refinement

*R*[*F* ^2^ > 2σ(*F* ^2^)] = 0.048
*wR*(*F* ^2^) = 0.143
*S* = 1.09
3255 reflections
236 parameters
H-atom parameters constrained
Δρ_max_ = 0.39 e Å^−3^
Δρ_min_ = −0.27 e Å^−3^

### 

Data collection: *SMART* (Bruker, 2000 ▶); cell refinement: *SAINT-Plus-NT* (Bruker, 2001 ▶); data reduction: *SAINT-Plus-NT*; program(s) used to solve structure: *SHELXTL-NT* (Sheldrick, 2008 ▶); program(s) used to refine structure: *SHELXTL-NT*; molecular graphics: *SHELXTL-NT*; software used to prepare material for publication: *PLATON* (Spek, 2009 ▶), *DIAMOND* (Bergerhoff *et al.*, 1996 ▶) and *publCIF* (Westrip, 2010 ▶).

## Supplementary Material

Crystal structure: contains datablocks I, global. DOI: 10.1107/S1600536810039279/jh2215sup1.cif

Structure factors: contains datablocks I. DOI: 10.1107/S1600536810039279/jh2215Isup2.hkl

Additional supplementary materials:  crystallographic information; 3D view; checkCIF report

## Figures and Tables

**Table 1:** Hydrogen-bond geometry (Å, °)

*D*—H⋯*A*	*D*—H	H⋯*A*	*D*⋯*A*	*D*—H⋯*A*
N2—H2*A*⋯O5^i^	0.86	1.91	2.767 (3)	177
O5—H5*A*⋯O2^ii^	0.84	2.10	2.889 (3)	157
C13—H13⋯O2^iii^	0.93	2.57	3.295 (4)	135
C14—H14*A*⋯O1^iv^	0.97	2.34	3.295 (3)	167
C17—H17*B*⋯O2^i^	0.96	2.57	3.466 (5)	155

Symmetry codes: (i) 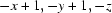; (ii) 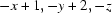; (iii) 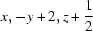; (iv) 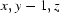.

**Fig. 1.**
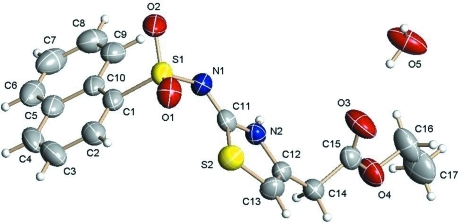
The molecular structure of (I) showing the atom-labelling scheme. Displacement ellipsoids are drawn at the 50% probability level and H atoms are shown as small spheres of arbitrary radius.

**Fig. 2.**
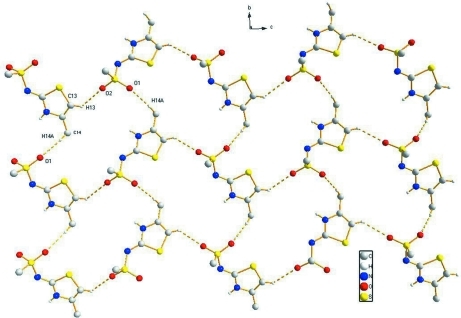
View of the C—H···O hydrogen bonds (dashed lines) in (I) to give an overall two-dimensional hydrogen-bonded network paralell to plane bc. H atoms not involved in hydrogen-bonding, as well as napthyl and ethoxy carbonylmethyl groups have been omitted for clarity.

**Fig. 3.**
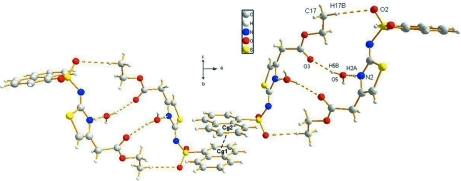
View of the dimeric hydrogen-bonding motif generated by C—H···O and O—H···O hydogen bonds (dashed lines) with cocrystallized water molecules. Also, the offset π···π [Cg1, Cg2i; symmetry code: (i) 1/2 - x, 1/2 + y, z] interaction is illustrated (dashed line).

## supplementary crystallographic information

### Comment

The biochemistry and pharmacology of sulfur containing compounds are a subject of intense current interest, especially from the point of view of public health. Obesity and diabetes are major causes of morbidity and mortality in many countries (Saiah, 2008). Excessive levels of glucocorticoids into the body can cause both metabolic complications. The regulation of glucocorticoid production involves two 11*b*-hydroxysteroid dehydrogenase (11*b*-HSD) isozymes, that interconvert cortisone and cortisol. 11*b*-HSD1 is a reductase that amplifies glucocorticoid action in a tissue-specific manner (Fotsch *et al.*, 2008).

Recent studies suggest that inhibition of 11*b*-HSD1 increases hepatic insulin sensivity along with decreased glucose production (Alberts *et al.* 2002). Selective inhibitors of 11*b*-HSD1 have considerable potential as treatments of type 2 diabetes and obesity (Vicker *et al.*, 2007). BVT 14225 is a new selective 11*b*-HSD1 inhibitor, it belongs to a class of arylsulfonamidothiazoles with *in vitro* and *in vivo* antidiabetic effects (Barf *et al.*, 2002).

In order to assist our knowledge about the electronic and steric requirements in arylsulfonamidothiazoles that show antidiabetic and antiobesity activities (Navarrete-Vázquez *et al.*, 2008), we have synthesized and determined the crystal structure of the title compound (I), which is a precursor in the synthesis of an amide BVT14225 bioisoster.

In the crystal structure of (I), the molecules are linked by intermolecular C—H···O hydrogen bonds to give an overall two-dimensional hydrogen-bonded network paralell to plane *bc* (Fig. 2, Table 1) (Desiraju & Steiner, 1999). The crystal structure is further stabilized by C—H···O and O—H···O hydogen bonds with cocrystallized water molecules, thus generating the dimeric hydrogen-bonding motif outlined in Fig. 3 (Table 1). In addition, adjacent naphthyl groups show offset π···π interactions (Fig. 3), with a distance between the centroids C1—C5–10, C5—C10 (*Cg*1, *Cg*2) of the naphtyl rings of 3.604 (2) Å (Hanton *et al.*, 1992; Adams *et al.*, 1996).

### Experimental

Naphthalene-1-sulfonylchloride (1.2 g, 0.0053 mol), was suspended in dry methylene chloride (10 ml) under nitrogen atmosphere. Triethylamine (0.9 ml, 0.0064 mol) was added slowly and stirred with a catalytic amount of 4-*N*,*N*-dimethylaminopyridine (0.1 eq). After 30 minutes, ethyl (2-amino-1,3-thiazol-4-yl)acetate (1 g, 0.0053 mol) was added dropwise, resulting in a brown solution. When all started material had been consumed, the solvent was removed *in vacuo*, the residue was neutralized with sodium bicarbonate. The precipitate resulting was filtered off to give a brown solid (m.p. 371 K). Single crystals of (I) were obtained from ethanol.

### Refinement

H atoms were positioned geometrically and constrained using the riding-model approximation [*C*-H_thiazolyl and naphtyl_ = 0.93 Å, *U*_iso_(H_thiazolyl and naphtyl_)= 1.2 *U*_eq_(C)]; [*C*-H_methylene_ = 0.97 Å, *U*_iso_(H_methylene_)= 1.2 *U*_eq_(C)], [*C*-H_methyl_ = 0.96 Å, *U*_iso_(H_methyl_)= 1.5 *U*_eq_(C)]. The hydrogen atoms bonded to O5 and N2 were located by difference Fourier maps. Its coordinates were refined with a distance restraint: O—H = 0.84 Å and [*U*_iso_(H) = 1.5 *U*_eq_(O)], N—H = 0.86 Å and [*U*_iso_(H) = 1.2 *U*_eq_(N)].

### Crystal data

**Table:**

C_17_H_16_N_2_O_4_S_2_·H_2_O	*D*_x_ = 1.419 Mg m^−^^3^
*M**_r_* = 394.45	Melting point: 371 K
Orthorhombic, *P**b**c**n*	Mo *K*α radiation, λ = 0.71073 Å
Hall symbol: -P 2n 2ab	Cell parameters from 7399 reflections
*a* = 29.582 (6) Å	θ = 2.6–23.6°
*b* = 7.9657 (17) Å	µ = 0.32 mm^−^^1^
*c* = 15.676 (3) Å	*T* = 273 K
*V* = 3694.0 (14) Å^3^	Rectangular prism, colourless
*Z* = 8	0.29 × 0.21 × 0.17 mm
*F*(000) = 1648	

### Data collection

**Table:**

Bruker SMART APEX CCD area detector diffractometer	3255 independent reflections
Radiation source: fine-focus sealed tube	2488 reflections with *I* > 2σ(*I*)
graphite	*R*_int_ = 0.056
Detector resolution: 8.3 pixels mm^-1^	θ_max_ = 25.0°, θ_min_ = 1.4°
phi and ω scans	*h* = −35→35
Absorption correction: multi-scan (*SADABS*; Sheldrick, 1996)	*k* = −9→9
*T*_min_ = 0.913, *T*_max_ = 0.948	*l* = −18→18
33131 measured reflections	

### Refinement

**Table:**

Refinement on *F*^2^	Primary atom site location: structure-invariant direct methods
Least-squares matrix: full	Secondary atom site location: difference Fourier map
*R*[*F*^2^ > 2σ(*F*^2^)] = 0.048	Hydrogen site location: inferred from neighbouring sites
*wR*(*F*^2^) = 0.143	H-atom parameters constrained
*S* = 1.09	*w* = 1/[σ^2^(*F*_o_^2^) + (0.0753*P*)^2^ + 1.2945*P*] where *P* = (*F*_o_^2^ + 2*F*_c_^2^)/3
3255 reflections	(Δ/σ)_max_ = 0.001
236 parameters	Δρ_max_ = 0.39 e Å^−^^3^
0 restraints	Δρ_min_ = −0.27 e Å^−^^3^

### Special details

**Table:**

Geometry. All e.s.d.'s (except the e.s.d. in the dihedral angle between two l.s. planes) are estimated using the full covariance matrix. The cell e.s.d.'s are taken into account individually in the estimation of e.s.d.'s in distances, angles and torsion angles; correlations between e.s.d.'s in cell parameters are only used when they are defined by crystal symmetry. An approximate (isotropic) treatment of cell e.s.d.'s is used for estimating e.s.d.'s involving l.s. planes.
Refinement. Refinement of *F*^2^ against ALL reflections. The weighted *R*-factor *wR* and goodness of fit *S* are based on *F*^2^, conventional *R*-factors *R* are based on *F*, with *F* set to zero for negative *F*^2^. The threshold expression of *F*^2^ > σ(*F*^2^) is used only for calculating *R*-factors(gt) *etc*. and is not relevant to the choice of reflections for refinement. *R*-factors based on *F*^2^ are statistically about twice as large as those based on *F*, and *R*- factors based on ALL data will be even larger.

### Fractional atomic coordinates and isotropic or equivalent isotropic displacement parameters (Å^2^)

**Table:**

	*x*	*y*	*z*	*U*_iso_*/*U*_eq_	
C1	0.30111 (9)	1.0099 (3)	−0.01807 (17)	0.0476 (7)	
C2	0.27124 (10)	1.0650 (4)	0.0426 (2)	0.0579 (8)	
H2	0.2818	1.1180	0.0915	0.069*	
C3	0.22466 (11)	1.0411 (4)	0.0306 (3)	0.0694 (9)	
H3	0.2044	1.0776	0.0719	0.083*	
C4	0.20921 (10)	0.9652 (4)	−0.0408 (2)	0.0679 (9)	
H4	0.1782	0.9534	−0.0487	0.081*	
C5	0.23901 (10)	0.9033 (4)	−0.1037 (2)	0.0562 (8)	
C6	0.22292 (12)	0.8195 (5)	−0.1765 (2)	0.0724 (10)	
H6	0.1919	0.8057	−0.1838	0.087*	
C7	0.25155 (14)	0.7585 (5)	−0.2363 (2)	0.0794 (11)	
H7	0.2402	0.7041	−0.2842	0.095*	
C8	0.29822 (13)	0.7776 (5)	−0.2257 (2)	0.0733 (9)	
H8	0.3178	0.7355	−0.2669	0.088*	
C9	0.31544 (11)	0.8571 (4)	−0.15595 (18)	0.0573 (7)	
H9	0.3466	0.8675	−0.1499	0.069*	
C10	0.28653 (9)	0.9240 (3)	−0.09264 (18)	0.0484 (7)	
C11	0.38848 (8)	0.8134 (3)	0.08417 (17)	0.0433 (6)	
C12	0.40857 (8)	0.5962 (3)	0.17449 (16)	0.0436 (6)	
C13	0.39714 (10)	0.7109 (4)	0.23161 (18)	0.0557 (7)	
H13	0.3984	0.6930	0.2902	0.067*	
C14	0.42320 (9)	0.4204 (3)	0.18915 (18)	0.0496 (7)	
H14A	0.4013	0.3457	0.1630	0.060*	
H14B	0.4230	0.3986	0.2500	0.060*	
C15	0.46919 (9)	0.3790 (4)	0.15489 (18)	0.0508 (7)	
C16	0.51735 (11)	0.1490 (5)	0.1211 (3)	0.0821 (11)	
H16A	0.5425	0.1802	0.1575	0.099*	
H16B	0.5228	0.1929	0.0643	0.099*	
C17	0.51244 (15)	−0.0349 (5)	0.1183 (3)	0.1036 (14)	
H17A	0.5089	−0.0774	0.1752	0.155*	
H17B	0.5389	−0.0835	0.0928	0.155*	
H17C	0.4863	−0.0637	0.0850	0.155*	
N1	0.38373 (7)	0.8826 (3)	0.00845 (14)	0.0499 (6)	
N2	0.40331 (7)	0.6559 (3)	0.09207 (13)	0.0428 (5)	
H2A	0.4093	0.5947	0.0483	0.051*	
O1	0.36124 (7)	1.1601 (2)	0.07509 (14)	0.0648 (6)	
O2	0.37576 (7)	1.1370 (3)	−0.07715 (14)	0.0639 (6)	
O3	0.49668 (8)	0.4789 (3)	0.13212 (19)	0.0858 (8)	
O4	0.47548 (7)	0.2156 (3)	0.15495 (15)	0.0698 (6)	
O5	0.57738 (8)	0.5513 (3)	0.04480 (16)	0.0876 (8)	
H5A	0.5842	0.6535	0.0484	0.131*	
H5B	0.5518	0.5450	0.0679	0.131*	
S1	0.35869 (2)	1.05939 (9)	−0.00081 (5)	0.0514 (2)	
S2	0.37963 (3)	0.89724 (10)	0.18608 (5)	0.0577 (3)	

### Atomic displacement parameters (Å^2^)

**Table:**

	*U*^11^	*U*^22^	*U*^33^	*U*^12^	*U*^13^	*U*^23^
C1	0.0426 (14)	0.0430 (14)	0.0572 (16)	−0.0020 (12)	−0.0018 (12)	0.0115 (13)
C2	0.0547 (18)	0.0499 (17)	0.0692 (19)	0.0008 (13)	0.0047 (15)	0.0069 (15)
C3	0.0510 (18)	0.063 (2)	0.094 (3)	0.0032 (15)	0.0181 (18)	0.0091 (19)
C4	0.0385 (15)	0.067 (2)	0.098 (3)	−0.0065 (14)	0.0017 (17)	0.023 (2)
C5	0.0482 (17)	0.0538 (17)	0.0665 (18)	−0.0140 (13)	−0.0068 (14)	0.0201 (15)
C6	0.062 (2)	0.074 (2)	0.082 (2)	−0.0258 (18)	−0.0198 (19)	0.026 (2)
C7	0.097 (3)	0.078 (2)	0.063 (2)	−0.034 (2)	−0.019 (2)	0.0146 (19)
C8	0.087 (3)	0.075 (2)	0.058 (2)	−0.0155 (19)	0.0034 (18)	0.0084 (17)
C9	0.0561 (17)	0.0624 (19)	0.0534 (17)	−0.0079 (14)	0.0026 (14)	0.0120 (15)
C10	0.0447 (15)	0.0450 (15)	0.0555 (16)	−0.0063 (12)	−0.0026 (13)	0.0184 (13)
C11	0.0314 (12)	0.0479 (15)	0.0505 (16)	−0.0008 (11)	−0.0009 (11)	−0.0038 (12)
C12	0.0347 (13)	0.0540 (17)	0.0422 (14)	0.0008 (11)	0.0002 (11)	0.0044 (12)
C13	0.0617 (17)	0.0663 (19)	0.0391 (15)	0.0072 (15)	0.0040 (13)	0.0001 (14)
C14	0.0413 (15)	0.0563 (17)	0.0512 (16)	0.0022 (12)	0.0036 (12)	0.0065 (13)
C15	0.0423 (15)	0.0605 (19)	0.0494 (16)	0.0001 (14)	−0.0031 (12)	−0.0024 (14)
C16	0.0498 (18)	0.092 (3)	0.105 (3)	0.0127 (17)	0.0076 (18)	−0.036 (2)
C17	0.088 (3)	0.086 (3)	0.137 (4)	0.028 (2)	0.028 (3)	−0.019 (3)
N1	0.0469 (13)	0.0547 (14)	0.0480 (13)	0.0037 (11)	−0.0023 (10)	0.0069 (11)
N2	0.0409 (12)	0.0480 (13)	0.0395 (11)	0.0024 (10)	0.0001 (9)	−0.0034 (9)
O1	0.0653 (14)	0.0492 (12)	0.0798 (15)	0.0002 (9)	−0.0171 (11)	−0.0087 (11)
O2	0.0493 (11)	0.0649 (13)	0.0774 (14)	−0.0120 (10)	−0.0017 (10)	0.0266 (11)
O3	0.0549 (13)	0.0784 (16)	0.124 (2)	−0.0084 (13)	0.0285 (13)	−0.0052 (15)
O4	0.0510 (12)	0.0629 (14)	0.0955 (17)	0.0114 (10)	0.0146 (11)	−0.0053 (12)
O5	0.0865 (17)	0.0755 (16)	0.1008 (18)	−0.0314 (13)	0.0476 (14)	−0.0389 (14)
S1	0.0429 (4)	0.0474 (4)	0.0641 (5)	−0.0047 (3)	−0.0060 (3)	0.0101 (3)
S2	0.0665 (5)	0.0547 (5)	0.0520 (4)	0.0092 (4)	0.0064 (3)	−0.0083 (3)

### Geometric parameters (Å, °)

**Table:**

C1—C2	1.370 (4)	C12—N2	1.385 (3)
C1—C10	1.421 (4)	C12—C14	1.484 (4)
C1—S1	1.769 (3)	C13—S2	1.727 (3)
C2—C3	1.404 (4)	C13—H13	0.9300
C2—H2	0.9300	C14—C15	1.499 (4)
C3—C4	1.352 (5)	C14—H14A	0.9700
C3—H3	0.9300	C14—H14B	0.9700
C4—C5	1.411 (5)	C15—O3	1.193 (3)
C4—H4	0.9300	C15—O4	1.315 (3)
C5—C6	1.406 (4)	C16—O4	1.448 (4)
C5—C10	1.426 (4)	C16—C17	1.473 (5)
C6—C7	1.353 (5)	C16—H16A	0.9700
C6—H6	0.9300	C16—H16B	0.9700
C7—C8	1.399 (5)	C17—H17A	0.9600
C7—H7	0.9300	C17—H17B	0.9600
C8—C9	1.363 (4)	C17—H17C	0.9600
C8—H8	0.9300	N1—S1	1.598 (2)
C9—C10	1.414 (4)	N2—H2A	0.8600
C9—H9	0.9300	O1—S1	1.437 (2)
C11—N1	1.316 (3)	O2—S1	1.439 (2)
C11—N2	1.335 (3)	O5—H5A	0.8399
C11—S2	1.751 (3)	O5—H5B	0.8400
C12—C13	1.323 (4)		
			
C2—C1—C10	121.9 (3)	C12—C13—H13	123.5
C2—C1—S1	116.3 (2)	S2—C13—H13	123.5
C10—C1—S1	121.7 (2)	C12—C14—C15	114.6 (2)
C1—C2—C3	119.8 (3)	C12—C14—H14A	108.6
C1—C2—H2	120.1	C15—C14—H14A	108.6
C3—C2—H2	120.1	C12—C14—H14B	108.6
C4—C3—C2	120.2 (3)	C15—C14—H14B	108.6
C4—C3—H3	119.9	H14A—C14—H14B	107.6
C2—C3—H3	119.9	O3—C15—O4	124.4 (3)
C3—C4—C5	121.5 (3)	O3—C15—C14	125.4 (3)
C3—C4—H4	119.2	O4—C15—C14	110.2 (2)
C5—C4—H4	119.2	O4—C16—C17	106.9 (3)
C6—C5—C4	121.4 (3)	O4—C16—H16A	110.3
C6—C5—C10	119.2 (3)	C17—C16—H16A	110.3
C4—C5—C10	119.4 (3)	O4—C16—H16B	110.3
C7—C6—C5	121.4 (3)	C17—C16—H16B	110.3
C7—C6—H6	119.3	H16A—C16—H16B	108.6
C5—C6—H6	119.3	C16—C17—H17A	109.5
C6—C7—C8	119.8 (3)	C16—C17—H17B	109.5
C6—C7—H7	120.1	H17A—C17—H17B	109.5
C8—C7—H7	120.1	C16—C17—H17C	109.5
C9—C8—C7	120.9 (3)	H17A—C17—H17C	109.5
C9—C8—H8	119.5	H17B—C17—H17C	109.5
C7—C8—H8	119.5	C11—N1—S1	120.0 (2)
C8—C9—C10	120.8 (3)	C11—N2—C12	116.5 (2)
C8—C9—H9	119.6	C11—N2—H2A	121.8
C10—C9—H9	119.6	C12—N2—H2A	121.8
C9—C10—C1	125.1 (3)	C15—O4—C16	118.9 (3)
C9—C10—C5	117.9 (3)	H5A—O5—H5B	104.1
C1—C10—C5	117.0 (3)	O1—S1—O2	115.50 (13)
N1—C11—N2	120.8 (2)	O1—S1—N1	113.12 (12)
N1—C11—S2	130.3 (2)	O2—S1—N1	106.95 (13)
N2—C11—S2	108.83 (19)	O1—S1—C1	107.55 (13)
C13—C12—N2	111.4 (2)	O2—S1—C1	107.85 (13)
C13—C12—C14	128.4 (2)	N1—S1—C1	105.29 (12)
N2—C12—C14	120.1 (2)	C13—S2—C11	90.25 (13)
C12—C13—S2	113.0 (2)		
			
C10—C1—C2—C3	−1.8 (4)	N2—C12—C14—C15	62.3 (3)
S1—C1—C2—C3	175.5 (2)	C12—C14—C15—O3	14.1 (4)
C1—C2—C3—C4	−0.5 (5)	C12—C14—C15—O4	−167.2 (2)
C2—C3—C4—C5	2.1 (5)	N2—C11—N1—S1	170.26 (19)
C3—C4—C5—C6	177.9 (3)	S2—C11—N1—S1	−11.7 (3)
C3—C4—C5—C10	−1.3 (4)	N1—C11—N2—C12	178.0 (2)
C4—C5—C6—C7	−179.3 (3)	S2—C11—N2—C12	−0.4 (3)
C10—C5—C6—C7	−0.1 (5)	C13—C12—N2—C11	0.4 (3)
C5—C6—C7—C8	0.4 (5)	C14—C12—N2—C11	177.6 (2)
C6—C7—C8—C9	0.0 (5)	O3—C15—O4—C16	−3.5 (5)
C7—C8—C9—C10	−0.7 (5)	C14—C15—O4—C16	177.7 (3)
C8—C9—C10—C1	−179.5 (3)	C17—C16—O4—C15	−172.3 (3)
C8—C9—C10—C5	0.9 (4)	C11—N1—S1—O1	26.1 (3)
C2—C1—C10—C9	−177.0 (3)	C11—N1—S1—O2	154.4 (2)
S1—C1—C10—C9	5.7 (4)	C11—N1—S1—C1	−91.0 (2)
C2—C1—C10—C5	2.5 (4)	C2—C1—S1—O1	−3.2 (2)
S1—C1—C10—C5	−174.7 (2)	C10—C1—S1—O1	174.1 (2)
C6—C5—C10—C9	−0.5 (4)	C2—C1—S1—O2	−128.4 (2)
C4—C5—C10—C9	178.6 (3)	C10—C1—S1—O2	48.9 (2)
C6—C5—C10—C1	179.9 (3)	C2—C1—S1—N1	117.7 (2)
C4—C5—C10—C1	−1.0 (4)	C10—C1—S1—N1	−65.0 (2)
N2—C12—C13—S2	−0.2 (3)	C12—C13—S2—C11	0.0 (2)
C14—C12—C13—S2	−177.1 (2)	N1—C11—S2—C13	−177.9 (3)
C13—C12—C14—C15	−121.0 (3)	N2—C11—S2—C13	0.27 (19)

### Hydrogen-bond geometry (Å, °)

**Table:**

*D*—H···*A*	*D*—H	H···*A*	*D*···*A*	*D*—H···*A*
N2—H2A···O5^i^	0.86	1.91	2.767 (3)	177
O5—H5A···O2^ii^	0.84	2.10	2.889 (3)	157
C13—H13···O2^iii^	0.93	2.57	3.295 (4)	135
C14—H14A···O1^iv^	0.97	2.34	3.295 (3)	167
C17—H17B···O2^i^	0.96	2.57	3.466 (5)	155

Symmetry codes: (i) −*x*+1, −*y*+1, −*z*; (ii) −*x*+1, −*y*+2, −*z*; (iii) *x*, −*y*+2, *z*+1/2; (iv) *x*, *y*−1, *z*.
